# Supplementary material for: Hyperactivity is linked to elevated cortisol levels: comprehensive behavioral analysis in the prenatal valproic acid-induced marmoset model of autism
Source: Transl Psychiatry. 2026 Jan 16;16:64. doi: 10.1038/s41398-025-03798-2 (PMC12873359; doi:10.1038/s41398-025-03798-2)

**Supplemental materials**

**Figure S1**: Average body weights (in grams) and age (weeks) of the adult subjects in the home-cage activity analysis. No significant between-group differences were detected (weight: *p* = 0.9634; age: *p* = 0.8541 for Student’s *t* test).


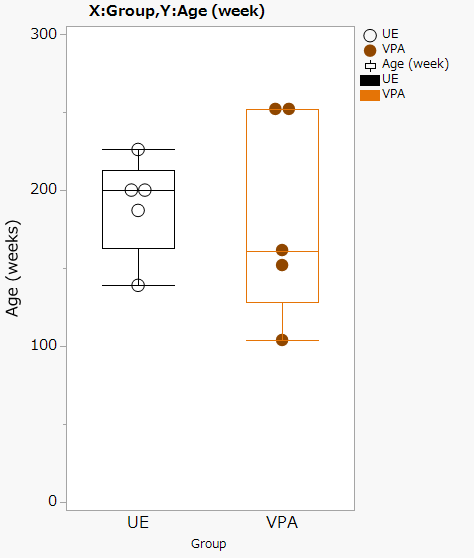

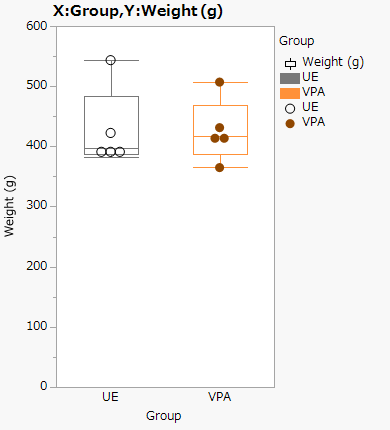

Supplement: Supplementary file 1 — Supplemental Figure S1 [file 41398_2025_3798_MOESM1_ESM.docx]
