# Supplementary material for: Hyperactivity is linked to elevated cortisol levels: comprehensive behavioral analysis in the prenatal valproic acid-induced marmoset model of autism
Source: Transl Psychiatry. 2026 Jan 16;16:64. doi: 10.1038/s41398-025-03798-2 (PMC12873359; doi:10.1038/s41398-025-03798-2)

**Supplemental materials**

**Figure S2**: Average weights (in grams) of the juvenile subjects in the 3-chamber test. There was no significant difference in weights between the two groups (*p* = 0.0878 for Student’s *t* test).


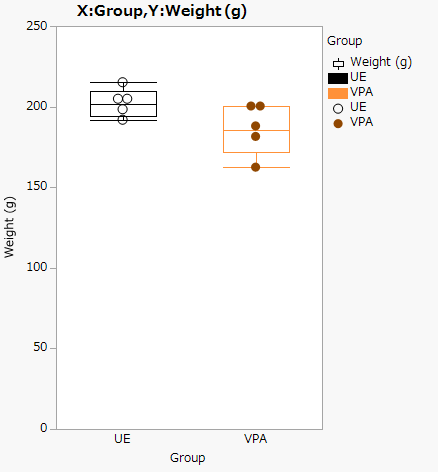

Supplement: Supplementary file 2 — Supplemental Figure S2 [file 41398_2025_3798_MOESM2_ESM.docx]
